# From inferring preferences to enabling choice: potentials of digital tools to improve substitute decision-making

**DOI:** 10.3389/fdgth.2026.1784416

**Published:** 2026-03-11

**Authors:** Florian Funer, Christin Hempeler

**Affiliations:** 1 Institute for Ethics and History of Medicine, Eberhard Karls University Tübingen, Tübingen, Germany; 2 Institute for Medical Ethics and History of Medicine, Ruhr University Bochum, Bochum, Germany; 3 Institute for History and Ethics of Medicine, Interdisciplinary Center for Health Sciences, Martin Luther University Halle-Wittenberg, Halle (Saale), Germany

**Keywords:** (Personalized) patient preference predictor (PPP/P4), advance care planning (ACP), advance directives (AD), artificial intelligence (AI), decision-making capacity, substituted judgment, surrogate decision-making

## Abstract

Respect for patient autonomy is a foundational principle in healthcare ethics, which holds that patients can make their own treatment decisions. However, sometimes patients lack the capacity to do so and surrogates must decide on their behalf in the sense of substitute decision-making. This is challenging, as guidance for these decisions is often lacking due to limited engagement in advance care planning (ACP) and the low prevalence of advance directives (ADs), which allow patients to pre-determine their treatment preferences. In response to these challenges, digital technologies employing artificial intelligence—particularly so-called (Personalized) Patient Preference Predictors (PPP or P4)—have recently received comprehensive scholarly attention, with initial studies exploring their technical feasibility. These tools aim to leverage AI's capacity to process large datasets to infer individual patients’ likely treatment preferences, thereby hoping to alleviate surrogates’ burden and to promote patient autonomy by facilitating treatment decisions more in line with patients’ preferences. In this article, we emphasize that autonomy is more robustly respected when substitute decisions rely on deliberate expressions of will formulated through ACP or documented in ADs rather than on even highly accurate predictions of treatment preferences. While we acknowledge the potential of PPPs/P4s to improve substitute decision-making when no explicit guidance exists, we caution against allowing current enthusiasm for AI-driven preference prediction to overlook the considerable potential that digital tools and AI offer for strengthening ACP and increasing completion of ADs. We therefore call for greater investment in using digital technologies to enhance ACP processes.

## 1 The challenges of substitute decision making and the promises of AI

Contemporary healthcare practice is grounded in an ethical commitment to respect patient autonomy, which holds that individuals should make their own healthcare decisions whenever possible (1, 2). However, there are situations in which patients are not or no longer able to do so, indicating a lack of decision-making capacity (3, 4). While acute and late-life care settings constitute paradigmatic contexts in which decision-making incapacity frequently arises, the need for substitute decision-making extends beyond these situations. Temporary incapacity due to, e.g., trauma, delirium or stroke, some neurological or mental health conditions raise comparable ethical challenges. Empirical studies show that approximately one quarter to one third of hospitalized adults lack decision-making capacity at the time key medical decisions are required (5, 6), and systematic reviews suggest that in psychiatric inpatient settings the prevalence of incapacity may be even higher, with weighted averages around 40%–45% of patients lacking decisional capacity for treatment decisions (7). Although the clinical dynamics and temporal horizons differ across contexts, the normative questions concerning how patient autonomy ought to be respected in the absence of contemporaneous decision-making capacity remain structurally similar.

When patients lack decision-making capacity, surrogates have to make decisions on their behalf through a process of substitute decision-making (8). They are typically expected to decide as the patient would have in the same situation. This, however, is no easy task. Ideally, patients have provided guidance for these situations, for example, by participating in advance-care planning (ACP) or documenting their treatment preferences in an advance directive (AD). ACP is a process in which patients discuss and reflect on their goals and values together with healthcare professionals to work out their preferences for future medical care (9–11). ADs are written documents that patients can prepare while they still have decision-making capacity to give instructions on by whom and how substituted decisions shall be made (4).

However, many patients do neither participate in ACP (12, 13) nor complete ADs (14–16), and even when they do, ADs are often vague or contextually inapplicable to guide the clinical decision at hand (17). As a result, surrogates are frequently left without clear indications of the patient's preferences and must attempt to reconstruct presumed wishes based on limited or indirect information. Surrogates often experience these situations as emotionally burdensome (18, 19), causing “stress, guilt over the decisions they made, and doubt regarding whether they had made the right decisions” (20, p. 336). Furthermore, research indicates that surrogates' assessment of the patient's presumed preference is often inaccurate. For example, a systematic review assessing the concordance of decision-making between a person and their surrogate in hypothetical treatment scenarios shows that surrogates misjudged their family members' treatment preferences in around one third of the cases (21).

In response, emerging digital solutions have been proposed that use methods of artificial intelligence (AI) to support substitute decision-making. In particular, so-called *(Personalized) Patient Preference Predictors* (PPP or P4) have recently received comprehensive scholarly attention (2, 22, 23), and first studies regarding their technical feasibility have been initiated (24–26). Such predictors aim to leverage AI to infer an individual patient's treatment preferences by analyzing large-scale data patterns. These tools are primarily designed to benefit patients and their surrogates: they are hoped to alleviate surrogates' decisional burden in absence of ADs and promote patient autonomy by facilitating treatment decisions more in line with patients' presumed preferences (2, 22, 23).

In this article, we assess to what extent the P4 can promote patient autonomy by predicting patients' treatment preferences. We argue that autonomy is more robustly respected when substitute decisions rely on deliberate expressions of will—formulated through ACP or documented in ADs—rather than on even highly accurate predictions of treatment preferences. While we acknowledge the potential of PPPs/P4s to improve substitute decision-making when no explicit guidance exists, we emphasize that the current enthusiasm for AI-driven preference prediction should not lead to overlooking the great potential digital tools and AI hold for strengthening ACP and increasing completion of ADs. In other words, the PPP/P4 offers a new solution to the question of how to make the best of situations in which explicit guidance for substitute decisions is lacking. Yet, equal effort should be devoted to ensuring such situations arise less often.

This paper presents a conceptual and normative analysis, grounded in a narrative review of the ethical, clinical, and technological literature on substitute decision-making, ADs and ACP, as well as AI-based decision support. The paper is structured as follows: In section 2, we outline the proposals of the PPP and P4 for using AI to infer patients' treatment preferences. In section 3, we draw on an established hierarchy of standards for substitute decision-making to highlight that the PPP/P4 might be useful to infer a patient's presumed preferences, yet, fostering ACP and the completion of ADs presents an ethically superior way to promote patient autonomy. Section 4 explores how digital technologies may help overcome current barriers of ACP and AD drafting, while also noting their risks and limitations. Finally, we conclude that digital support for explicit expressions of will is ethically more compelling than focusing on ever more sophisticated preference prediction alone. Therefore, predictive tools should not become the primary focus of technological innovation but potentials of digital tools and AI to strengthen ACP and completion of ADs ought also be considered.

## 2 (Personalized) patient preference prediction—a suitable digital solution?

While the conceptual roots of the *Patient Preference Predictor* (PPP) date back to 2007 (27),^1^ the idea is usually attributed to Annette Rid and David Wendler, who articulated a formal proposal in 2014—prior to the widespread adoption of AI (22, 23). Their proposal builds on empirical evidence indicating that treatment preferences correlate with certain individual characteristics, such as age, gender, ethnicity, marital status, geographical location, education, occupational status, and religious affiliation (22). They hence proposed that a well-designed statistical model could leverage these predictors to infer an individual's most likely treatment preference in cases of decisional incapacity.

To generate the necessary data, Rid and Wendler suggested conducting large-scale surveys that would collect individuals' demographic characteristics, data on their physical, psychological and social status, general attitudes and values, relevant personal experiences, and preferences for various hypothetical medical scenarios (22, 23). By comparing this reference data to the characteristics of incapacitated patients, such a model could yield probabilistic estimates of patient preferences for specific medical treatment decisions (22, 23).^2^ According to Rid and Wendler, evidence suggests that a PPP would be able to predict preferences for an “*average* person” at least as accurately as surrogates (22, p. 112), and that many patients would endorse its integration into the substitute decision-making process (29). The authors envisioned three possible roles for the PPP in clinical practice: (i) as a discussion aid for clinicians and surrogates; (ii) as a “weak default” to guide decisions unless a surrogate objects; or (iii) as a “strong default” to be adhered to unless “compelling reason” suggest that the predicted treatment preference contradicts the patient's values (22, p. 115).

Building on this foundation, more recent proposals have explored ways to enhance patient preference prediction using AI. Lamanna and Byrne, for example, introduced the so-called “autonomy algorithm” (30)—a machine-learning system that could mine digital data from electronic health records (EHRs) and social media activity (e.g., “likes” for specific contents) to estimate the likelihood that a patient would consent to a specific treatment (30). Assuming a good performance, it was proposed that in cases where no legally authorized surrogate exists, decision-making authority should be assigned to an “autonomy algorithm” rather than to family members (31).

Most recently, Earp and colleagues proposed an advanced and even more individualized model: the *“Personalized Patient Preference Predictor*” (P4) (2). Rather than relying on population-level correlations, the P4 uses patient-specific digital data to train a large language model (LLM) that effectively serves as a “digital psychological twin” (p. 15), which could be asked about the patient's treatment preferences when necessary. This LLM would be fine-tuned^3^ using materials generated by the patient—such as emails, blog posts, or social media content—or information documented in EHRs, in which patients' values and preferences may be implicitly encoded. Additionally, patients could be asked to express their preferences for hypothetical scenarios during routine care or ACP conversations or even be “incentivized to participate in specially designed, value-eliciting discrete choice experiments” (p. 16). Depending on the availability and type of personal data, multiple versions of P4 could be developed (2), varying in how well they can substantiate statements about preferences. Importantly, Earp et al. highlight that the impact of different data sources could be weighted according to patients' wishes for their inclusion, as well as their strength in producing accurate predictions.

In summary, both PPP and P4 attempt to infer a patient's preferences to make substitute decisions in the absence of explicit guidance. The proposals have sparked extensive debate, encompassing an appraisal of their benefits as well as a range of criticisms. Benefits include the aspiration to approximate incapacitated patients' treatment preferences more systematically than existing surrogate-based approaches, thereby promoting respect for patient autonomy, and the potential to assist surrogate decision-makers by offering additional decision-relevant information in high-burden clinical situations (2, 22). Concerns include skepticism about their technical feasibility (32–35), development efforts and costs (36), practical risks such as AI-safety and alignment problems associated with incentive structures (35, 37), environmental impact (36) as well as issues about the model like its predictive accuracy (37–39), explainability (40), potential biases and data quality (36, 38–45), and how to properly validate it (35, 42, 43), for example in terms of clinical improvement in goal-concordant care and a decline in decision regret (33). Moreover, it has been questioned whether the PPP or P4 would truly reduce surrogate burden or might even exacerbate it (28, 46, 47).^4^

Commentators additionally highlighted an important limitation of PPP/P4s: their generation of probabilistic reconstructions of likely preferences rather than expressions of a patient's will (32, 36, 46, 48). Even when predictions draw on extensive patient-specific digital traces, they remain inferential outputs based on correlations and pattern recognition. As such, they differ categorically from preferences that patients have deliberately formed, reflected upon, and explicitly authorized for use in future decision-making contexts. This has direct ethical implications and will be central to the assessment of whether and how such tools can meaningfully contribute to respecting patient autonomy. This will be investigated in more detail in the following section.

## 3 Can the prediction of patient preferences support respect for autonomy?

One central aim of the PPP and its personalized successor, the P4, is to promote patient autonomy. Proponents of the PPP/P4 appeal to the so-called *substituted judgment standard*, a well-established guide to substitute decision-making. According to this standard, surrogates can respect the autonomy of a now incompetent patient by making the decision the patient would have made under present circumstances if they were competent—that is, by “reconstructing […] the autonomous decision [they] would have made if [they] were able” (4). The PPP and P4 seek to improve the accuracy of this reconstruction by identifying patients' hypothetical treatment preferences, thereby arguably promoting autonomy by increasing alignment between surrogate decisions and patients' preferences (22, 49, 50).

Since the publication of the PPP/P4 proposals, however, critics have questioned whether these tools can genuinely advance patient autonomy. A recurring concern is that accurately identifying patients' treatment preferences is not, by itself, sufficient for respecting autonomy (38, 49, 50). Several authors have, for example, argued that the decision about patients' presumed treatment preferences ought to be reached for the right reasons, for example, reasons that the patient themself would have endorsed (32, 51–54) or taking into account a person's deeper, higher-order values, which the PPP/P4 cannot accomplish (34, 46). Similarly, it has been emphasized that patients may have second-order preferences regarding *how* their presumed treatment preferences are determined (55). Moreover, commentators have stressed the importance of relational aspects and dialogical engagement in substitute decision-making, which the PPP/P4 cannot replace (33, 46, 56). Finally, several commentators have cautioned against relying on social media data as indicators of treatment preferences, noting that opinions expressed there may be socially shaped (57) and that the digital self may differ from the individual's authentic self (42, 45).

Recently, Earp and colleagues clarified their position on the role of the P4 in promoting patient autonomy (58). They agree with their critics that “respect for autonomy is not simply reducible to mere preference satisfaction” (p. 462). Instead, they highlight that “respect for autonomy in healthcare exists on a spectrum” (p. 463), which corresponds to the widely accepted hierarchy of standards for substitute decision-making (4). This distinguishes between the above-described *substituted judgement standard*, grounded in the principle of respect for patient autonomy, and the *best interest standard*, which derives from the principle of beneficence and guides surrogates to choose what would benefit the patient most, based on what a reasonable person would prefer under current circumstances (4, 59).

**Figure 1:**
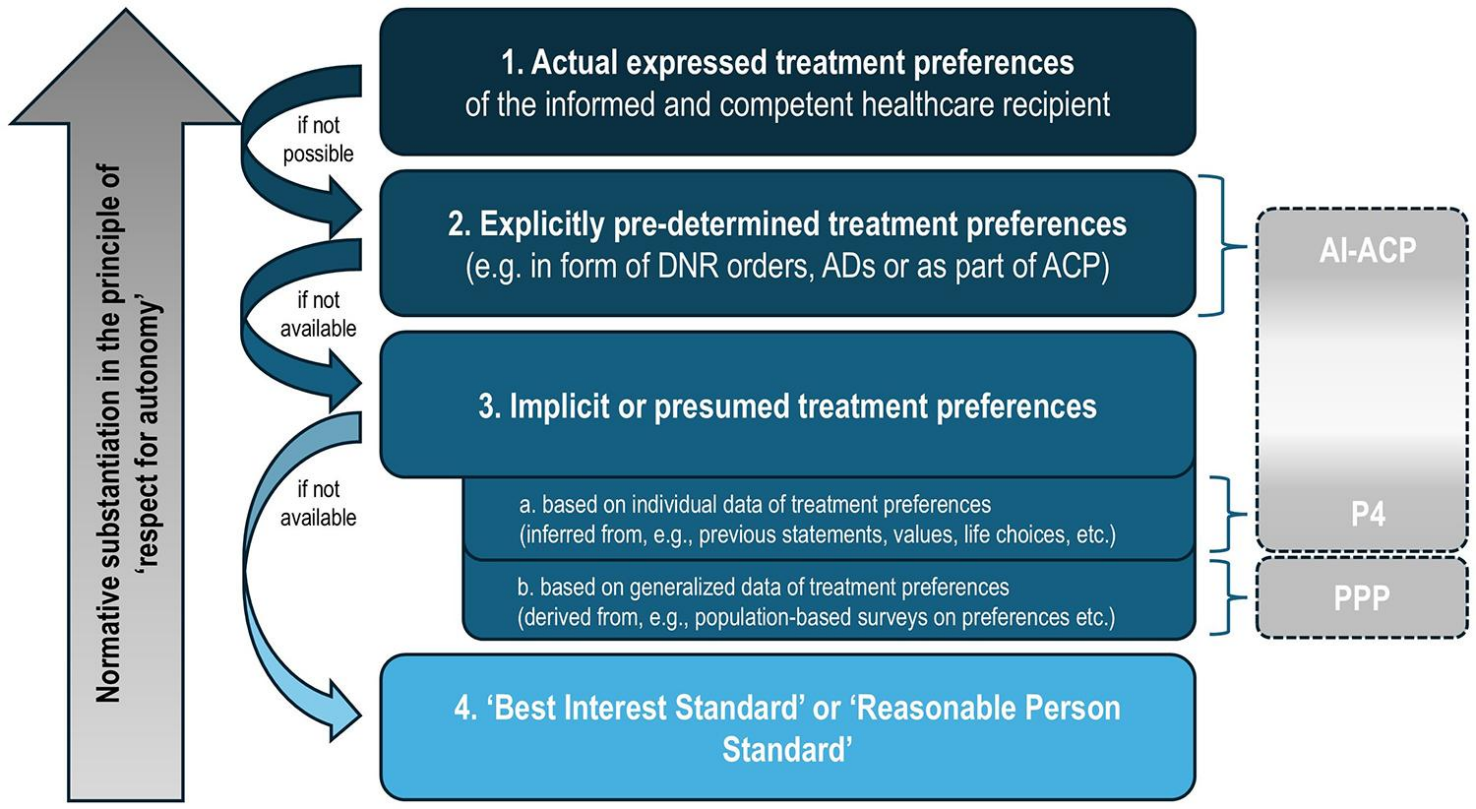
Hierarchy for (substitute) decision-making in healthcare and corresponding hierarchy of proposed digital support tools.

Given the value typically afforded to patient autonomy, these two standards result in the following hierarchy for substitute decision-making (see Figure 1): First, if possible, surrogates should rely on the patient's *previously expressed* treatment preferences—ideally documented in an AD or conveyed verbally during ACP. Second, when such information is not available, surrogates are expected to make a decision consistent with the patient's *presumed* treatment preference, determined based on the patient's values, prior life choices and/or statements on related topics. Third, if a patient has never been competent, or if no information on their preferences or values exists, surrogate decisions should be based on the best interest standard (4, 59).

Figure 1 illustrates this widely accepted hierarchy, highlighting both the normative priority of explicitly expressed patient preferences (level 2) before implicit or presumed treatment preferences (level 3) and the relative positioning of digital approaches to support substitute decision-making. As can be seen, the PPP and P4 represent innovative methods for modeling a patient's *presumed* preferences (level 3) as these predictors try to infer patients' treatment preferences from various data sources.^5^ However, the hierarchy shows that autonomy is more robustly respected when decisions are grounded in deliberate expressions of will during ACP or documented in ADs (level 2)—as acknowledged by proponents of the PPP and P4 (2, 22, 58). There are two main reasons for that.

The first is *epistemic*: substitute decisions based on explicitly pre-determined treatment preferences—whether expressed during ACP or documented in AD—are supported by direct evidence of preferences the patient has actually formed and expressed. As such, they provide comparatively strong grounds for confidence that the resulting decision aligns with choice the patient would have made if competent. By contrast, as several commentators have emphasized (32, 36, 46, 48), the preference indications generated by PPPs/P4s constitute probabilistic inferences. Even when highly accurate at the aggregate level, such inferences remain indirect and epistemically weaker, as they do not constitute evidence of the patient's own deliberated preferences but rather predictions about what the patient is statistically likely to prefer.^6^

The second reason is *normative*: Buchanan and Brock (59) importantly highlight that “[s]ometimes advance directives are mistakenly viewed as one especially important kind of *evidence* concerning what the individual would choose. The error here is that of failing to see that advance directives are *performative*, not merely evidentiary” (p. 115 f., emphasis adopted). What they emphasize here is that autonomy is more robustly respected when substitute decisions rely on explicitly pre-determined treatment preferences rather than presumed preferences because the explicit expression of autonomous choices—especially when emerging from ACP—involves deliberative decision-making and thus constitutes an act of self-determination. Buchanan and Brock (59) therefore conclude that “evidence concerning *preferences*, especially if it is indirect and inferential, does not have the same moral weight as evidence of *deliberate choice*. [..] The contemporaneous choice of a competent individual under conditions of informed consent is an act of will, not the mere expression of a preference” (p. 116, emphases adopted).

Considering the epistemic and normative superiority of basing substitute decisions on explicitly expressed patient preferences over inferred ones, we wish to emphasize that current enthusiasm for AI-driven preference prediction should not lead to overlooking the great potential digital tools and AI hold for strengthening ACP and increasing completion of ADs. Especially if, as Mertes suggests, the effort required to achieve a satisfactory predictive accuracy for the P4 may exceed the efforts required for facilitating the implementation of ADs (36), then there are strong practical and ethical reasons to give priority to the latter, without denying that the PPP/P4 may be useful for situations where this approach remains unsuccessful.^7^ Against this background, in the following section, we will show how the rise of digital decision aids, automated documentation systems, and AI-supported platforms offer new opportunities to improve the quality and availability of ACP and ADs (61–64).

## 4 Digital strategies to support advance care planning

As outlined in the previous section, explicitly expressed preferences offer a more robust epistemic and normative foundation for substitute decision-making than presumed (inferred) preferences. Yet, ADs and ACP remain underutilized in practice—despite their normative superiority and widely acknowledged benefits, such as preventing unwanted medical treatment, improving the quality of end-of-life care, and strengthening patient autonomy (11, 65). This raises an urgent question: how can we better support individuals in articulating and documenting their values and treatment wishes while they still have decision-making capacity?

Over the past decade, efforts to develop interventions to address barriers to ACP implementation and completion of ADs have increased considerably in both number and variety (61). Yet, the empirical record is mixed: many ACP initiatives have struggled with low uptake, limited depth of reflection, suboptimal timing relative to disease trajectories, and variable integration into clinical workflows (66–68). In our view, however, these limitations do not undermine the normative importance of ACP as such, but they underscore the need for more supportive, accessible and dynamically revisable forms of ACP—an area in which digital and AI-assisted tools may have genuine added value.

Especially the evolution of ACP delivery methods, shifting from booklets and individual counseling to multi-media and web-based digital technologies, has offered promise for mitigating many of the existing barriers (61–64). Although there has been considerable development recently, Gloeckler and colleagues (62) importantly highlight that “[t]here is much unrealized potential in the enrichment of advance directives with digital technology” (p. 350). In what follows, we therefore explore how digital tools—and AI—may be harnessed to reduce key barriers and limitations associated with ADs and ACP, thereby helping to realize their full potential. To this end, Table 1 outlines some prominent barriers to ADs/ACP and how digital technologies may help to mitigate them. While not exhaustive, this discussion aims to stimulate further inquiry into the field. We recognize that this approach has risks and limitations, which we discuss at the end of this section.

**Table 1:** Overview of commonly reported barriers to advance care planning (ACP) and advance directives (AD) on a structural, practical, provider, and patient level and the potential of digital tools to mitigate them.

Main barriers of ACP and ADs	Digital strategy for mitigation	Examples of existing tools^8^
Structural level		
Limited healthcare resources (e.g., time, staff) (66, 69–72)	Patient-facing ACP tools reduce clinician time by preparing patients in advance and enabling a more targeted provider involvement.	“PREPARE for Your Care” (73, 74), “PreCare” (75)
Inequitable access to ACP (76)	Web-based platforms increase accessibility across time and location; tools can be tailored for marginalized populations.	“Explore Your Preferences For Treatment And Care” (64, 77), “Making Your Wishes Known: Planning Your Medical Future” (78, 79)
Practical level		
Format of advance directives (ADs): predominantly text-based, lengthy, and complex (76, 80)	Use of dialogical options, apps, visualizations, video or game-based formats, and assistive tech (e.g., text-to-speech or speech-to-text) improves accessibility and comprehensibility.	“DiAD app” (81, 82), “Accordons-nous” (83)
Storage and access to ACP documentation/ADs (66)	Integration with electronic health records (EHRs) or secure digital platforms enables timely, centralized access—potentially across care settings.	“EPaCCS” (84), “MyDirectives” (85), “US Advance Care Plan Registry” (86)
Poor quality or limited applicability of ACP/AD content (66, 80)	Digital tools can improve document quality by offering structured templates, proactively highlighting inconsistencies/conflicts, simulating (future) clinical scenarios, and supporting iterative refinement based on the patient- and illness-specific context.	“DiAD app” (81, 82), “PREPARE for Your Care” (73, 74), “MyDirectives” (85)
Provider level		
Reluctance to discuss ACP related issues (70–72), e.g., due to fear of eroding patient hope (66)	Digital decision aids and structured guides can lower the threshold for initiating ACP conversations and provide procedural support to clinicians.	EHR prompts to start conversation (87)
Limited provider knowledge, skills, or experience in ACP (66, 69–72)	Educational modules, interactive FAQs, and chatbots can support clinicians by guiding them through the ACP process and addressing common knowledge gaps.	Chatbot ACP-Trainer (88), “Synthetic Patients” (89), VR Teaching Modules (90)
Uncertainty about “the right moment” to initiate ACP (66, 91)	EHR-interventions can assist in identifying appropriate time for ACP discussions based on patient data and send timely prompts to clinicians.	EHR prompts to start conversation (87), Machine Learning-based Mortality Model Notifications (92)
Patient level		
Patients with reduced decision-making capacity (70, 72)	Serve as an aid to supported decision-making, e.g., through recipient-oriented communication and more small-scale guidance through relevant questions/aspects, potentially involving family members.	Tailored ACP websites, e.g., for people with dementia (93, 94), “Our Memory Care Wishes” (95), digital visual aids (96)
Reluctance of patients to engage with future care (66, 69–72)	Peer testimonials, engaging media formats, gamification, and accessible design lower psychological barriers to ACP engagement.	“Anticip’action” (97)

First approaches to leverage digital technologies for ACP aimed to address widespread practical barriers for ACP, mainly the timely and easy access to ADs (66, 67). Electronic systems for storing and sharing ACP preferences (98) and consumer-driven digital platforms (99) enhance availability and patient engagement (e.g., the “US Advance Care Plan Registry”, “MyDirectives”) (98–104). Integration into EHRs supports standardized documentation and timely clinical access (100–102). Further approaches focus on improved care coordination, like the Electronic Palliative Care Coordination Systems (EPaCCS), which facilitates the electronic exchange of information among healthcare professionals across institutions (103). Sometimes the use of EHR interventions goes beyond storage and access, for example, by including structured documentation templates (e.g., for do-not-resuscitate orders or preferred places of care and death) to improve the quality of ADs, as well as automated alerts based on patient age or diagnosis to prompt healthcare professionals to initiate ACP discussions (100).^9^ These interventions help address further barriers to ACP, such as the poor quality or inapplicability of many ADs (66, 80) and healthcare providers' difficulties to identify an appropriate time for ACP (66, 91). Emerging evidence highlights that such EHR-based interventions can indeed enhance ACP engagement and outcomes (100, 102).

A subsequent wave of technological advancements to digitally improve ACP focused on lowering the threshold for accessing ACP and enhancing patients' health literacy as well as knowledge about and engagement with ACP, which often constitute barriers to successful ACP (69–72, 76, 93). Various web-based tools, including interactive websites, web-based portals or apps (e.g., “PREPARE for Your Care”, “PROVEN”, “Making Your Wishes Known: Planning Your Medical Future”) (61), have been developed as decision aids to reach a broad audience and offer users flexible access in terms of time and location (64).^10^ Such tools may further be designed for populations with specific diseases or be culturally adapted (61), thus facilitating access of minoritized groups (107).

Tools like “PREPARE for your care” (prepareforyourcare.org) (108, 109) include components such as exploring personal values and treatment preferences, facilitating communication with healthcare providers or family members, and enabling the creation of preference documentation (64, 106). The DiAD app (81, 82) similarly supports users in clarifying their goals of care through the integration of the “advance care compass”, which simulates future quality-of-life scenarios to help users reflect on and digitally record their preferences.^11^ Digital ACP tools may also include video or game-based elements (61), text-to-speech options, and peer testimonials, which are elements valued by prospective users, such as people living with dementia (110). A scoping review on the feasibility and effectiveness of web-based ACP programs reported high user satisfaction and effectiveness in enhancing ACP-related knowledge, improving communication, and increasing documentation (64). Furthermore, digital decision-aids seem to foster shared decision-making between patients, their family members, and healthcare professionals (111). Despite this progress, a comprehensive review of ACP-apps in the US found that overall apps are currently “limited in both quality and scope” (p. 988), highlighting significant unrealized potential in this domain (112).

Recent advancements in AI, particularly LLMs, present novel opportunities to further reduce barriers in ACP through more personalized, adaptive, and context-sensitive interventions. Current innovations increasingly focus on enhancing informational accessibility, supporting decision-making, and personalizing AD content. LLM-based systems, for example, can facilitate dynamic, conversational interfaces tailored to users' individual situations and needs (113). Predictive analytics embedded in such tools can offer evidence-based insights into treatment options and their likely outcomes, thereby fostering reflection and dialogue in shared decision-making processes and during the creation of ADs (114). LLMs can also help identify relevant information on key ACP domains such as goals of care, preferences regarding life-sustaining treatment, and palliative care options within EHRs (115) and prompt health professionals to initiate ACP conversations on the preselected or associated topics. Furthermore, interactive LLM-driven ACP tools, which allow users to virtually choose courses of action and anticipate the associated consequences, may enhance individuals' “ability to vividly project themselves into potential future healthcare scenarios, leading to more likely hypothetical picture of the respondents future care goals and preferences” (116, p. 169). Moreover, LLMs can adapt language complexity within seconds to improve comprehension of medical information, increasing accessibility for a wide range of individuals with varying levels of understanding or cognitive ability. AI-powered chatbots and digital companions are also being explored as scalable tools to facilitate ACP discussions, particularly among populations less likely to engage with traditional care planning resources (117).

Within this repertoire of digital strategies to enhance ACP, we certainly recognize a potential application for a P4 (2). For example, preference predictions could function as reflective prompts, encouraging individuals to consider their personal values and preferences in the context of specific decisions. An illustrative application of this concept of authorizing predictions is the “resuscitation decision algorithm,” (118) which synthesizes personal attributes (e.g., age, health status, general values) and compares them with population-level preferences regarding resuscitation to generate individualized statements, such as: “Individuals who are similar to you with regard to characteristics A, B, C would choose ‘CPR yes’ in x% of cases as long as the likelihood for survival-to-discharge was over 30%” (118, p. 182). Similarly, the P4 could be used to forecast likely preferences, presenting them to users for review, acceptance, or modification—thereby integrating preference predictions into the ACP process as a form of decision support (33, 122, 123).

Finally, any enthusiasm for digitally enhanced ACP must be tempered by careful consideration of its potential risks and limitations. Regarding risks, it should be noted that AI-driven conversational agents and decision aids can introduce new forms of bias, opacity, and manipulation (119). They may nudge users towards certain formulations of values or treatment choices, over-simplify complex trade-offs, or generate hallucinated information that patients and clinicians mistakenly treat as reliable (120). If AI comes to mediate much of the ACP process, safeguards will be needed to ensure that it facilitates, rather than impedes, genuine deliberation and that it supports, rather than undermines, the first-person authority of patients over their own future care. Moreover, overreliance on AI-mediated ACP risks attenuating the dialogical and relational dimensions of care planning central to many accounts of autonomy.

Furthermore, there are also limitations to enhancing ACP and completion of ADs through digital technologies and AI that need to be acknowledged. While we have shown that digital technologies provide new opportunities to address existing barriers to successful implementation of ACP, there are barriers—particularly those on a societal and structural level—that lie beyond the reach of digital interventions. For example, low public awareness (76) or that talking about illness, dying, and death remain a societal taboo (66) are significant obstacles that technology alone cannot overcome. Moreover, for any ACP intervention to be successful, certain structural preconditions such as institutional support and financial resources are required (67).

Given these limitations, we must recognize that—despite the potential to improve the uptake of ACP and the completion of ADs through digital technologies and AI—situations in which explicit guidance for substitute decision-making is unavailable will continue to exist. For these situations, predictive tools like the PPP/P4 can play a valuable role in approximating patients' presumed preferences. Our argument, therefore, is not against the development or use of PPPs/P4s. Rather, we advance the more modest claim that these systems should not become the primary focus of technological innovation but that potentials of digital tools and AI to strengthen ACP and completion of ADs ought also to be promoted.

## 5 Conclusion

The use of (Personalized) Patient Preference Predictors has been proposed as an innovative solution to the persistent difficulties of substitute decision-making in healthcare, particularly given the limited engagement with ACP and the low prevalence and quality of ADs. These tools aim to leverage AI's capacity to process large datasets to infer individual patients' likely treatment preferences, thereby hoping to alleviate surrogates' burden and to promote patient autonomy by facilitating treatment decisions more in line with patients' presumed preferences. We highlighted that, even if PPP/P4 were to achieve high levels of predictive accuracy, substitute decisions grounded in explicitly pre-determined choices—articulated through ACP processes and documented in ADs—remain epistemically and normatively distinctive. They do not merely provide information about what a patient would likely prefer; they constitute acts of self-determination that authorize others to act on one's behalf.

Against this background, we advocated that current enthusiasm for AI-driven preference prediction should not lead to overlooking the great potential digital tools and AI offer for strengthening ACP and increasing completion of ADs. We provided an overview of how purpose-built digital tools for ACP hold promise for supporting patients in understanding complex information, reflecting on their values, documenting their wishes, and revisiting these over time. If designed with appropriate safeguards, digital tools may thus help address some of the well-known barriers and limitations of ACP. At the same time, the growing appeal of AI-based predictive systems carries the risk of diverting attention, resources, and institutional commitment away from these efforts. While PPP/P4 may offer a response to situations in which explicit guidance is lacking, their exploration should remain clearly subordinate to, and not come at the expense of, sustained investment in improving ACP practice—so that such situations arise less frequently in the first place.

## Data Availability

The original contributions presented in the study are included in the article, further inquiries can be directed to the corresponding author.

## Footnotes

^1^Shalowitz, Garrett-Mayer and Wendler called it the “Population-Based Treatment Indicator” (27).

^2^Ditto and Clark suggest using disease-specific PPPs, incorporating data from people having experience with the respective disease (28).

^3^The fine-tuned LLM could use either a generic LLM as its basis or “one trained on population-level data” (p. 16) like the PPP. Fine-tuning, as envisioned by Earp et al., consists of modifying the final layers of an LLM’s neural network by training it on a specific text corpus that comes from or describes the individual (2).

^4^Beyond the concerns raised directly in response to PPP/P4 proposals, many of the more general challenges associated with deploying AI-based systems in high-stakes clinical contexts also apply to the use of PPP/P4, for example, adulteration of source data, vulnerability to adversial inputs, model drift over time, and cybersecurity threats. For responses to some of these concerns, see (121).

^5^Some versions of the P4 might go beyond merely trying to identify a patient’s presumed preferences, e.g., when the sources of data largely comprise explicit indications of treatment preferences. Furthermore, Dresser expresses uncertainty whether the PPP would provide information on presumed preferences or rather on best interests (44).

^6^Some may object that—assuming a high predictive accuracy of the PPP/P4—decisions based on ACP or ADs may not actually be epistemically superior to those informed by the P4, particularly given research that indicates patients’ ability to anticipate how health changes will affect their assessment of quality of life is limited (60). This objection, however, is misleading: on many versions of the P4, preference predictions would be equally constrained by individuals’ limited capacities to foresee their future life circumstances. During ACP, physicians may help correct certain biases—for example, regarding living with certain illnesses—an opportunity that is unavailable if preference predictions are generated from data produced without adequate explanation or guidance.

^7^There may also be concern that directing resources and attention toward the development of PPP/P4 could risk undermining efforts to increase update of ACP, thereby potentially reinforcing the very conditions that make predictive tools appear attractive.

^9^While most of these tools target patients, some also aim to improve healthcare providers’ knowledge about ACP (106).

^10^While the DiAD app does not use AI, its founders highlight that its underlying methods, which mainly consist of relative quantification and logical deductions, offer “intrinsic compatibility with [AI]” (81).

^11^Göcking and colleagues similarly suggest that “preference-sensitive decision timelines” may be integrated into EHRs to give guidance on when to initiate ACP (105).
